# Azimuthal separation in nearly back-to-back jet topologies in inclusive 2- and 3-jet events in ${\text {p}} {\text {p}}$ collisions at $\sqrt{s}=13\,\text {Te}\text {V}$

**DOI:** 10.1140/epjc/s10052-019-7276-4

**Published:** 2019-09-18

**Authors:** A. M. Sirunyan, A. Tumasyan, W. Adam, F. Ambrogi, E. Asilar, T. Bergauer, J. Brandstetter, E. Brondolin, M. Dragicevic, J. Erö, A. Escalante Del Valle, M. Flechl, R. Frühwirth, V. M. Ghete, J. Hrubec, M. Jeitler, N. Krammer, I. Krätschmer, D. Liko, T. Madlener, I. Mikulec, N. Rad, H. Rohringer, J. Schieck, R. Schöfbeck, M. Spanring, D. Spitzbart, A. Taurok, W. Waltenberger, J. Wittmann, C.-E. Wulz, M. Zarucki, V. Chekhovsky, V. Mossolov, J. Suarez Gonzalez, E. A. De Wolf, D. Di Croce, X. Janssen, J. Lauwers, M. Pieters, M. Van De Klundert, H. Van Haevermaet, P. Van Mechelen, N. Van Remortel, S. Abu Zeid, F. Blekman, J. D’Hondt, I. De Bruyn, J. De Clercq, K. Deroover, G. Flouris, D. Lontkovskyi, S. Lowette, I. Marchesini, S. Moortgat, L. Moreels, Q. Python, K. Skovpen, S. Tavernier, W. Van Doninck, P. Van Mulders, I. Van Parijs, D. Beghin, B. Bilin, H. Brun, B. Clerbaux, G. De Lentdecker, H. Delannoy, B. Dorney, G. Fasanella, L. Favart, R. Goldouzian, A. Grebenyuk, A. K. Kalsi, T. Lenzi, J. Luetic, N. Postiau, E. Starling, L. Thomas, C. Vander Velde, P. Vanlaer, D. Vannerom, Q. Wang, T. Cornelis, D. Dobur, A. Fagot, M. Gul, I. Khvastunov, D. Poyraz, C. Roskas, D. Trocino, M. Tytgat, W. Verbeke, B. Vermassen, M. Vit, N. Zaganidis, H. Bakhshiansohi, O. Bondu, S. Brochet, G. Bruno, P. David, C. Delaere, M. Delcourt, B. Francois, A. Giammanco, G. Krintiras, V. Lemaitre, A. Magitteri, A. Mertens, M. Musich, K. Piotrzkowski, A. Saggio, M. Vidal Marono, S. Wertz, J. Zobec, F. L. Alves, G. A. Alves, L. Brito, G. Correia Silva, C. Hensel, A. Moraes, M. E. Pol, P. Rebello Teles, E. Belchior Batista Das Chagas, W. Carvalho, J. Chinellato, E. Coelho, E. M. Da Costa, G. G. Da Silveira, D. De Jesus Damiao, C. De Oliveira Martins, S. Fonseca De Souza, H. Malbouisson, D. Matos Figueiredo, M. Melo De Almeida, C. Mora Herrera, L. Mundim, H. Nogima, W. L. Prado Da Silva, L. J. Sanchez Rosas, A. Santoro, A. Sznajder, M. Thiel, E. J. Tonelli Manganote, F. Torres Da Silva De Araujo, A. Vilela Pereira, S. Ahuja, C. A. Bernardes, L. Calligaris, T. R. Fernandez Perez Tomei, E. M. Gregores, P. G. Mercadante, S. F. Novaes, SandraS. Padula, D. Romero Abad, A. Aleksandrov, R. Hadjiiska, P. Iaydjiev, A. Marinov, M. Misheva, M. Rodozov, M. Shopova, G. Sultanov, A. Dimitrov, L. Litov, B. Pavlov, P. Petkov, W. Fang, X. Gao, L. Yuan, M. Ahmad, J. G. Bian, G. M. Chen, H. S. Chen, M. Chen, Y. Chen, C. H. Jiang, D. Leggat, H. Liao, Z. Liu, F. Romeo, S. M. Shaheen, A. Spiezia, J. Tao, C. Wang, Z. Wang, E. Yazgan, H. Zhang, J. Zhao, Y. Ban, G. Chen, A. Levin, J. Li, L. Li, Q. Li, Y. Mao, S. J. Qian, D. Wang, Z. Xu, Y. Wang, C. Avila, A. Cabrera, C. A. Carrillo Montoya, L. F. Chaparro Sierra, C. Florez, C. F. González Hernández, M. A. Segura Delgado, B. Courbon, N. Godinovic, D. Lelas, I. Puljak, T. Sculac, Z. Antunovic, M. Kovac, V. Brigljevic, D. Ferencek, K. Kadija, B. Mesic, A. Starodumov, T. Susa, M. W. Ather, A. Attikis, G. Mavromanolakis, J. Mousa, C. Nicolaou, F. Ptochos, P. A. Razis, H. Rykaczewski, M. Finger, M. Finger, E. Ayala, E. Carrera Jarrin, A. Ellithi Kamel, M. A. Mahmoud, E. Salama, A. Carvalho Antunes De Oliveira, R. K. Dewanjee, K. Ehataht, M. Kadastik, M. Raidal, C. Veelken, P. Eerola, H. Kirschenmann, J. Pekkanen, M. Voutilainen, J. Havukainen, J. K. Heikkilä, T. Järvinen, V. Karimäki, R. Kinnunen, T. Lampén, K. Lassila-Perini, S. Laurila, S. Lehti, T. Lindén, P. Luukka, T. Mäenpää, H. Siikonen, E. Tuominen, J. Tuominiemi, T. Tuuva, M. Besancon, F. Couderc, M. Dejardin, D. Denegri, J. L. Faure, F. Ferri, S. Ganjour, A. Givernaud, P. Gras, G. Hamel de Monchenault, P. Jarry, C. Leloup, E. Locci, J. Malcles, G. Negro, J. Rander, A. Rosowsky, M. Ö. Sahin, M. Titov, A. Abdulsalam, C. Amendola, I. Antropov, F. Beaudette, P. Busson, C. Charlot, R. Granier de Cassagnac, I. Kucher, S. Lisniak, A. Lobanov, J. Martin Blanco, M. Nguyen, C. Ochando, G. Ortona, P. Pigard, R. Salerno, J. B. Sauvan, Y. Sirois, A. G. Stahl Leiton, A. Zabi, A. Zghiche, J.-L. Agram, J. Andrea, D. Bloch, J.-M. Brom, E. C. Chabert, V. Cherepanov, C. Collard, E. Conte, J.-C. Fontaine, D. Gelé, U. Goerlach, M. Jansová, A.-C. Le Bihan, N. Tonon, P. Van Hove, S. Gadrat, S. Beauceron, C. Bernet, G. Boudoul, N. Chanon, R. Chierici, D. Contardo, P. Depasse, H. El Mamouni, J. Fay, L. Finco, S. Gascon, M. Gouzevitch, G. Grenier, B. Ille, F. Lagarde, I. B. Laktineh, H. Lattaud, M. Lethuillier, L. Mirabito, A. L. Pequegnot, S. Perries, A. Popov, V. Sordini, M. Vander Donckt, S. Viret, S. Zhang, A. Khvedelidze, Z. Tsamalaidze, C. Autermann, L. Feld, M. K. Kiesel, K. Klein, M. Lipinski, M. Preuten, M. P. Rauch, C. Schomakers, J. Schulz, M. Teroerde, B. Wittmer, V. Zhukov, A. Albert, D. Duchardt, M. Endres, M. Erdmann, T. Esch, R. Fischer, S. Ghosh, A. Güth, T. Hebbeker, C. Heidemann, K. Hoepfner, H. Keller, S. Knutzen, L. Mastrolorenzo, M. Merschmeyer, A. Meyer, P. Millet, S. Mukherjee, T. Pook, M. Radziej, H. Reithler, M. Rieger, F. Scheuch, A. Schmidt, D. Teyssier, G. Flügge, O. Hlushchenko, B. Kargoll, T. Kress, A. Künsken, T. Müller, A. Nehrkorn, A. Nowack, C. Pistone, O. Pooth, H. Sert, A. Stahl, M. Aldaya Martin, T. Arndt, C. Asawatangtrakuldee, I. Babounikau, K. Beernaert, O. Behnke, U. Behrens, A. Bermúdez Martínez, D. Bertsche, A. A. Bin Anuar, K. Borras, V. Botta, A. Campbell, P. Connor, C. Contreras-Campana, F. Costanza, V. Danilov, A. De Wit, M. M. Defranchis, C. Diez Pardos, D. Domínguez Damiani, G. Eckerlin, T. Eichhorn, A. Elwood, E. Eren, E. Gallo, A. Geiser, J. M. Grados Luyando, A. Grohsjean, P. Gunnellini, M. Guthoff, M. Haranko, A. Harb, J. Hauk, H. Jung, M. Kasemann, J. Keaveney, C. Kleinwort, J. Knolle, D. Krücker, W. Lange, A. Lelek, T. Lenz, K. Lipka, W. Lohmann, R. Mankel, I.-A. Melzer-Pellmann, A. B. Meyer, M. Meyer, M. Missiroli, G. Mittag, J. Mnich, V. Myronenko, S. K. Pflitsch, D. Pitzl, A. Raspereza, M. Savitskyi, P. Saxena, P. Schütze, C. Schwanenberger, R. Shevchenko, A. Singh, N. Stefaniuk, H. Tholen, A. Vagnerini, G. P. Van Onsem, R. Walsh, Y. Wen, K. Wichmann, C. Wissing, O. Zenaiev, R. Aggleton, S. Bein, L. Benato, A. Benecke, V. Blobel, M. Centis Vignali, T. Dreyer, E. Garutti, D. Gonzalez, J. Haller, A. Hinzmann, A. Karavdina, G. Kasieczka, R. Klanner, R. Kogler, N. Kovalchuk, S. Kurz, V. Kutzner, J. Lange, D. Marconi, J. Multhaup, M. Niedziela, D. Nowatschin, A. Perieanu, A. Reimers, O. Rieger, C. Scharf, P. Schleper, S. Schumann, J. Schwandt, J. Sonneveld, H. Stadie, G. Steinbrück, F. M. Stober, M. Stöver, D. Troendle, A. Vanhoefer, B. Vormwald, M. Akbiyik, C. Barth, M. Baselga, S. Baur, E. Butz, R. Caspart, T. Chwalek, F. Colombo, W. De Boer, A. Dierlamm, N. Faltermann, B. Freund, M. Giffels, M. A. Harrendorf, F. Hartmann, S. M. Heindl, U. Husemann, F. Kassel, I. Katkov, S. Kudella, H. Mildner, S. Mitra, M. U. Mozer, Th. Müller, M. Plagge, G. Quast, K. Rabbertz, M. Schröder, I. Shvetsov, G. Sieber, H. J. Simonis, R. Ulrich, S. Wayand, M. Weber, T. Weiler, S. Williamson, C. Wöhrmann, R. Wolf, G. Anagnostou, G. Daskalakis, T. Geralis, A. Kyriakis, D. Loukas, G. Paspalaki, I. Topsis-Giotis, G. Karathanasis, S. Kesisoglou, P. Kontaxakis, A. Panagiotou, N. Saoulidou, E. Tziaferi, K. Vellidis, K. Kousouris, I. Papakrivopoulos, G. Tsipolitis, I. Evangelou, C. Foudas, P. Gianneios, P. Katsoulis, P. Kokkas, S. Mallios, N. Manthos, I. Papadopoulos, E. Paradas, J. Strologas, F. A. Triantis, D. Tsitsonis, M. Bartók, M. Csanad, N. Filipovic, P. Major, M. I. Nagy, G. Pasztor, O. Surányi, G. I. Veres, G. Bencze, C. Hajdu, D. Horvath, Á. Hunyadi, F. Sikler, T. Á. Vámi, V. Veszpremi, G. Vesztergombi, N. Beni, S. Czellar, J. Karancsi, A. Makovec, J. Molnar, Z. Szillasi, P. Raics, Z. L. Trocsanyi, B. Ujvari, S. Choudhury, J. R. Komaragiri, P. C. Tiwari, S. Bahinipati, C. Kar, P. Mal, K. Mandal, A. Nayak, D. K. Sahoo, S. K. Swain, S. Bansal, S. B. Beri, V. Bhatnagar, S. Chauhan, R. Chawla, N. Dhingra, R. Gupta, A. Kaur, A. Kaur, M. Kaur, S. Kaur, R. Kumar, P. Kumari, M. Lohan, A. Mehta, K. Sandeep, S. Sharma, J. B. Singh, G. Walia, A. Bhardwaj, B. C. Choudhary, R. B. Garg, M. Gola, S. Keshri, Ashok Kumar, S. Malhotra, M. Naimuddin, P. Priyanka, K. Ranjan, Aashaq Shah, R. Sharma, R. Bhardwaj, M. Bharti, R. Bhattacharya, S. Bhattacharya, U. Bhawandeep, D. Bhowmik, S. Dey, S. Dutt, S. Dutta, S. Ghosh, K. Mondal, S. Nandan, A. Purohit, P. K. Rout, A. Roy, S. Roy Chowdhury, S. Sarkar, M. Sharan, B. Singh, S. Thakur, P. K. Behera, R. Chudasama, D. Dutta, V. Jha, V. Kumar, P. K. Netrakanti, L. M. Pant, P. Shukla, T. Aziz, M. A. Bhat, S. Dugad, G. B. Mohanty, N. Sur, B. Sutar, RavindraKumar Verma, S. Banerjee, S. Bhattacharya, S. Chatterjee, P. Das, M. Guchait, Sa. Jain, S. Karmakar, S. Kumar, M. Maity, G. Majumder, K. Mazumdar, N. Sahoo, T. Sarkar, S. Chauhan, S. Dube, V. Hegde, A. Kapoor, K. Kothekar, S. Pandey, A. Rane, S. Sharma, S. Chenarani, E. Eskandari Tadavani, S. M. Etesami, M. Khakzad, M. Mohammadi Najafabadi, M. Naseri, F. Rezaei Hosseinabadi, B. Safarzadeh, M. Zeinali, M. Felcini, M. Grunewald, M. Abbrescia, C. Calabria, A. Colaleo, D. Creanza, L. Cristella, N. De Filippis, M. De Palma, A. Di Florio, F. Errico, L. Fiore, A. Gelmi, G. Iaselli, S. Lezki, G. Maggi, M. Maggi, G. Miniello, S. My, S. Nuzzo, A. Pompili, G. Pugliese, R. Radogna, A. Ranieri, A. Sharma, L. Silvestris, R. Venditti, P. Verwilligen, G. Zito, G. Abbiendi, C. Battilana, D. Bonacorsi, L. Borgonovi, S. Braibant-Giacomelli, R. Campanini, P. Capiluppi, A. Castro, F. R. Cavallo, S. S. Chhibra, C. Ciocca, G. Codispoti, M. Cuffiani, G. M. Dallavalle, F. Fabbri, A. Fanfani, P. Giacomelli, C. Grandi, L. Guiducci, F. Iemmi, S. Marcellini, G. Masetti, A. Montanari, F. L. Navarria, A. Perrotta, F. Primavera, A. M. Rossi, T. Rovelli, G. P. Siroli, N. Tosi, S. Albergo, A. Di Mattia, R. Potenza, A. Tricomi, C. Tuve, G. Barbagli, K. Chatterjee, V. Ciulli, C. Civinini, R. D’Alessandro, E. Focardi, G. Latino, P. Lenzi, M. Meschini, S. Paoletti, L. Russo, G. Sguazzoni, D. Strom, L. Viliani, L. Benussi, S. Bianco, F. Fabbri, D. Piccolo, F. Ferro, F. Ravera, E. Robutti, S. Tosi, A. Benaglia, A. Beschi, L. Brianza, F. Brivio, V. Ciriolo, S. Di Guida, M. E. Dinardo, S. Fiorendi, S. Gennai, A. Ghezzi, P. Govoni, M. Malberti, S. Malvezzi, A. Massironi, D. Menasce, L. Moroni, M. Paganoni, D. Pedrini, S. Ragazzi, T. Tabarelli de Fatis, S. Buontempo, N. Cavallo, A. Di Crescenzo, F. Fabozzi, F. Fienga, G. Galati, A. O. M. Iorio, W. A. Khan, L. Lista, S. Meola, P. Paolucci, C. Sciacca, E. Voevodina, P. Azzi, N. Bacchetta, D. Bisello, A. Boletti, A. Bragagnolo, R. Carlin, P. Checchia, M. Dall’Osso, P. De Castro Manzano, T. Dorigo, U. Dosselli, F. Gasparini, U. Gasparini, A. Gozzelino, S. Lacaprara, P. Lujan, M. Margoni, A. T. Meneguzzo, P. Ronchese, R. Rossin, F. Simonetto, A. Tiko, E. Torassa, M. Zanetti, P. Zotto, G. Zumerle, A. Braghieri, A. Magnani, P. Montagna, S. P. Ratti, V. Re, M. Ressegotti, C. Riccardi, P. Salvini, I. Vai, P. Vitulo, L. Alunni Solestizi, M. Biasini, G. M. Bilei, C. Cecchi, D. Ciangottini, L. Fanò, P. Lariccia, E. Manoni, G. Mantovani, V. Mariani, M. Menichelli, A. Rossi, A. Santocchia, D. Spiga, K. Androsov, P. Azzurri, G. Bagliesi, L. Bianchini, T. Boccali, L. Borrello, R. Castaldi, M. A. Ciocci, R. Dell’Orso, G. Fedi, L. Giannini, A. Giassi, M. T. Grippo, F. Ligabue, E. Manca, G. Mandorli, A. Messineo, F. Palla, A. Rizzi, P. Spagnolo, R. Tenchini, G. Tonelli, A. Venturi, P. G. Verdini, L. Barone, F. Cavallari, M. Cipriani, N. Daci, D. Del Re, E. Di Marco, M. Diemoz, S. Gelli, E. Longo, B. Marzocchi, P. Meridiani, G. Organtini, F. Pandolfi, R. Paramatti, F. Preiato, S. Rahatlou, C. Rovelli, F. Santanastasio, N. Amapane, R. Arcidiacono, S. Argiro, M. Arneodo, N. Bartosik, R. Bellan, C. Biino, N. Cartiglia, F. Cenna, S. Cometti, M. Costa, R. Covarelli, N. Demaria, B. Kiani, C. Mariotti, S. Maselli, E. Migliore, V. Monaco, E. Monteil, M. Monteno, M. M. Obertino, L. Pacher, N. Pastrone, M. Pelliccioni, G. L. Pinna Angioni, A. Romero, M. Ruspa, R. Sacchi, K. Shchelina, V. Sola, A. Solano, D. Soldi, A. Staiano, S. Belforte, V. Candelise, M. Casarsa, F. Cossutti, G. Della Ricca, F. Vazzoler, A. Zanetti, D. H. Kim, G. N. Kim, M. S. Kim, J. Lee, S. Lee, S. W. Lee, C. S. Moon, Y. D. Oh, S. Sekmen, D. C. Son, Y. C. Yang, H. Kim, D. H. Moon, G. Oh, J. Goh, T. J. Kim, S. Cho, S. Choi, Y. Go, D. Gyun, S. Ha, B. Hong, Y. Jo, K. Lee, K. S. Lee, S. Lee, J. Lim, S. K. Park, Y. Roh, H. S. Kim, J. Almond, J. Kim, J. S. Kim, H. Lee, K. Lee, K. Nam, S. B. Oh, B. C. Radburn-Smith, S. h. Seo, U. K. Yang, H. D. Yoo, G. B. Yu, D. Jeon, H. Kim, J. H. Kim, J. S. H. Lee, I. C. Park, Y. Choi, C. Hwang, J. Lee, I. Yu, V. Dudenas, A. Juodagalvis, J. Vaitkus, I. Ahmed, Z. A. Ibrahim, M. A. B. Md Ali, F. Mohamad Idris, W. A. T. Wan Abdullah, M. N. Yusli, Z. Zolkapli, H. Castilla-Valdez, E. De La Cruz-Burelo, M. C. Duran-Osuna, I. Heredia-De La Cruz, R. Lopez-Fernandez, J. Mejia Guisao, R. I. Rabadan-Trejo, G. Ramirez-Sanchez, R Reyes-Almanza, A. Sanchez-Hernandez, S. Carrillo Moreno, C. Oropeza Barrera, F. Vazquez Valencia, J. Eysermans, I. Pedraza, H. A. Salazar Ibarguen, C. Uribe Estrada, A. Morelos Pineda, D. Krofcheck, S. Bheesette, P. H. Butler, A. Ahmad, M. Ahmad, M. I. Asghar, Q. Hassan, H. R. Hoorani, A. Saddique, M. A. Shah, M. Shoaib, M. Waqas, H. Bialkowska, M. Bluj, B. Boimska, T. Frueboes, M. Górski, M. Kazana, K. Nawrocki, M. Szleper, P. Traczyk, P. Zalewski, K. Bunkowski, A. Byszuk, K. Doroba, A. Kalinowski, M. Konecki, J. Krolikowski, M. Misiura, M. Olszewski, A. Pyskir, M. Walczak, P. Bargassa, C. Beirão Da Cruz E Silva, A. Di Francesco, P. Faccioli, B. Galinhas, M. Gallinaro, J. Hollar, N. Leonardo, L. Lloret Iglesias, M. V. Nemallapudi, J. Seixas, G. Strong, O. Toldaiev, D. Vadruccio, J. Varela, A. Golunov, I. Golutvin, V. Karjavin, V. Korenkov, G. Kozlov, A. Lanev, A. Malakhov, V. Matveev, V. V. Mitsyn, P. Moisenz, V. Palichik, V. Perelygin, S. Shmatov, S. Shulha, V. Smirnov, V. Trofimov, B. S. Yuldashev, A. Zarubin, V. Zhiltsov, V. Golovtsov, Y. Ivanov, V. Kim, E. Kuznetsova, P. Levchenko, V. Murzin, V. Oreshkin, I. Smirnov, D. Sosnov, V. Sulimov, L. Uvarov, S. Vavilov, A. Vorobyev, Yu. Andreev, A. Dermenev, S. Gninenko, N. Golubev, A. Karneyeu, M. Kirsanov, N. Krasnikov, A. Pashenkov, D. Tlisov, A. Toropin, V. Epshteyn, V. Gavrilov, N. Lychkovskaya, V. Popov, I. Pozdnyakov, G. Safronov, A. Spiridonov, A. Stepennov, V. Stolin, M. Toms, E. Vlasov, A. Zhokin, T. Aushev, M. Chadeeva, P. Parygin, D. Philippov, S. Polikarpov, E. Popova, V. Rusinov, V. Andreev, M. Azarkin, I. Dremin, M. Kirakosyan, S. V. Rusakov, A. Terkulov, A. Baskakov, A. Belyaev, E. Boos, M. Dubinin, L. Dudko, A. Ershov, A. Gribushin, V. Klyukhin, O. Kodolova, I. Lokhtin, I. Miagkov, S. Obraztsov, S. Petrushanko, V. Savrin, A. Snigirev, V. Blinov, T. Dimova, L. Kardapoltsev, D. Shtol, Y. Skovpen, I. Azhgirey, I. Bayshev, S. Bitioukov, D. Elumakhov, A. Godizov, V. Kachanov, A. Kalinin, D. Konstantinov, P. Mandrik, V. Petrov, R. Ryutin, S. Slabospitskii, A. Sobol, S. Troshin, N. Tyurin, A. Uzunian, A. Volkov, A. Babaev, S. Baidali, P. Adzic, P. Cirkovic, D. Devetak, M. Dordevic, J. Milosevic, J. Alcaraz Maestre, A. Álvarez Fernández, I. Bachiller, M. Barrio Luna, J. A. Brochero Cifuentes, M. Cerrada, N. Colino, B. De La Cruz, A. Delgado Peris, C. Fernandez Bedoya, J. P. Fernández Ramos, J. Flix, M. C. Fouz, O. Gonzalez Lopez, S. Goy Lopez, J. M. Hernandez, M. I. Josa, D. Moran, A. Pérez-Calero Yzquierdo, J. Puerta Pelayo, I. Redondo, L. Romero, M. S. Soares, A. Triossi, C. Albajar, J. F. de Trocóniz, J. Cuevas, C. Erice, J. Fernandez Menendez, S. Folgueras, I. Gonzalez Caballero, J. R. González Fernández, E. Palencia Cortezon, V. Rodríguez Bouza, S. Sanchez Cruz, P. Vischia, J. M. Vizan Garcia, I. J. Cabrillo, A. Calderon, B. Chazin Quero, J. Duarte Campderros, M. Fernandez, P. J. Fernández Manteca, A. García Alonso, J. Garcia-Ferrero, G. Gomez, A. Lopez Virto, J. Marco, C. Martinez Rivero, P. Martinez Ruiz del Arbol, F. Matorras, J. Piedra Gomez, C. Prieels, T. Rodrigo, A. Ruiz-Jimeno, L. Scodellaro, N. Trevisani, I. Vila, R. Vilar Cortabitarte, D. Abbaneo, B. Akgun, E. Auffray, P. Baillon, A. H. Ball, D. Barney, J. Bendavid, M. Bianco, A. Bocci, C. Botta, T. Camporesi, M. Cepeda, G. Cerminara, E. Chapon, Y. Chen, G. Cucciati, D. d’Enterria, A. Dabrowski, V. Daponte, A. David, A. De Roeck, N. Deelen, M. Dobson, T. du Pree, M. Dünser, N. Dupont, A. Elliott-Peisert, P. Everaerts, F. Fallavollita, D. Fasanella, G. Franzoni, J. Fulcher, W. Funk, D. Gigi, A. Gilbert, K. Gill, F. Glege, M. Guilbaud, D. Gulhan, J. Hegeman, V. Innocente, A. Jafari, P. Janot, O. Karacheban, J. Kieseler, A. Kornmayer, M. Krammer, C. Lange, P. Lecoq, C. Lourenço, L. Malgeri, M. Mannelli, F. Meijers, J. A. Merlin, S. Mersi, E. Meschi, P. Milenovic, F. Moortgat, M. Mulders, J. Ngadiuba, S. Orfanelli, L. Orsini, F. Pantaleo, L. Pape, E. Perez, M. Peruzzi, A. Petrilli, G. Petrucciani, A. Pfeiffer, M. Pierini, F. M. Pitters, D. Rabady, A. Racz, T. Reis, G. Rolandi, M. Rovere, H. Sakulin, C. Schäfer, C. Schwick, M. Seidel, M. Selvaggi, A. Sharma, P. Silva, P. Sphicas, A. Stakia, J. Steggemann, M. Tosi, D. Treille, A. Tsirou, V. Veckalns, W. D. Zeuner, L. Caminada, K. Deiters, W. Erdmann, R. Horisberger, Q. Ingram, H. C. Kaestli, D. Kotlinski, U. Langenegger, T. Rohe, S. A. Wiederkehr, M. Backhaus, L. Bäni, P. Berger, N. Chernyavskaya, G. Dissertori, M. Dittmar, M. Donegà, C. Dorfer, C. Grab, C. Heidegger, D. Hits, J. Hoss, T. Klijnsma, W. Lustermann, R. A. Manzoni, M. Marionneau, M. T. Meinhard, F. Micheli, P. Musella, F. Nessi-Tedaldi, J. Pata, F. Pauss, G. Perrin, L. Perrozzi, S. Pigazzini, M. Quittnat, D. Ruini, D. A. Sanz Becerra, M. Schönenberger, L. Shchutska, V. R. Tavolaro, K. Theofilatos, M. L. Vesterbacka Olsson, R. Wallny, D. H. Zhu, T. K. Aarrestad, C. Amsler, D. Brzhechko, M. F. Canelli, A. De Cosa, R. Del Burgo, S. Donato, C. Galloni, T. Hreus, B. Kilminster, I. Neutelings, D. Pinna, G. Rauco, P. Robmann, D. Salerno, K. Schweiger, C. Seitz, Y. Takahashi, A. Zucchetta, Y. H. Chang, K. y. Cheng, T. H. Doan, Sh. Jain, R. Khurana, C. M. Kuo, W. Lin, A. Pozdnyakov, S. S. Yu, P. Chang, Y. Chao, K. F. Chen, P. H. Chen, W.-S. Hou, Arun Kumar, Y. y. Li, R.-S. Lu, E. Paganis, A. Psallidas, A. Steen, J. f. Tsai, B. Asavapibhop, N. Srimanobhas, N. Suwonjandee, A. Bat, F. Boran, S. Cerci, S. Damarseckin, Z. S. Demiroglu, F. Dolek, C. Dozen, I. Dumanoglu, E. Eskut, S. Girgis, G. Gokbulut, Y. Guler, E. Gurpinar, I. Hos, C. Isik, E. E. Kangal, O. Kara, A. Kayis Topaksu, U. Kiminsu, M. Oglakci, G. Onengut, K. Ozdemir, S. Ozturk, A. Polatoz, U. G. Tok, S. Turkcapar, I. S. Zorbakir, C. Zorbilmez, B. Isildak, G. Karapinar, M. Yalvac, M. Zeyrek, I. O. Atakisi, E. Gülmez, M. Kaya, O. Kaya, S. Tekten, E. A. Yetkin, M. N. Agaras, S. Atay, A. Cakir, K. Cankocak, Y. Komurcu, S. Sen, B. Grynyov, L. Levchuk, F. Ball, L. Beck, J. J. Brooke, D. Burns, E. Clement, D. Cussans, O. Davignon, H. Flacher, J. Goldstein, G. P. Heath, H. F. Heath, L. Kreczko, D. M. Newbold, S. Paramesvaran, B. Penning, T. Sakuma, D. Smith, V. J. Smith, J. Taylor, A. Titterton, K. W. Bell, A. Belyaev, C. Brew, R. M. Brown, D. Cieri, D. J. A. Cockerill, J. A. Coughlan, K. Harder, S. Harper, J. Linacre, E. Olaiya, D. Petyt, C. H. Shepherd-Themistocleous, A. Thea, I. R. Tomalin, T. Williams, W. J. Womersley, G. Auzinger, R. Bainbridge, P. Bloch, J. Borg, S. Breeze, O. Buchmuller, A. Bundock, S. Casasso, D. Colling, L. Corpe, P. Dauncey, G. Davies, M. Della Negra, R. Di Maria, Y. Haddad, G. Hall, G. Iles, T. James, M. Komm, C. Laner, L. Lyons, A.-M. Magnan, S. Malik, A. Martelli, J. Nash, A. Nikitenko, V. Palladino, M. Pesaresi, A. Richards, A. Rose, E. Scott, C. Seez, A. Shtipliyski, T. Strebler, S. Summers, A. Tapper, K. Uchida, T. Virdee, N. Wardle, D. Winterbottom, J. Wright, S. C. Zenz, J. E. Cole, P. R. Hobson, A. Khan, P. Kyberd, C. K. Mackay, A. Morton, I. D. Reid, L. Teodorescu, S. Zahid, K. Call, J. Dittmann, K. Hatakeyama, H. Liu, C. Madrid, B. Mcmaster, N. Pastika, C. Smith, R. Bartek, A. Dominguez, A. Buccilli, S. I. Cooper, C. Henderson, P. Rumerio, C. West, D. Arcaro, T. Bose, D. Gastler, D. Rankin, C. Richardson, J. Rohlf, L. Sulak, D. Zou, G. Benelli, X. Coubez, D. Cutts, M. Hadley, J. Hakala, U. Heintz, J. M. Hogan, K. H. M. Kwok, E. Laird, G. Landsberg, J. Lee, Z. Mao, M. Narain, J. Pazzini, S. Piperov, S. Sagir, R. Syarif, E. Usai, D. Yu, R. Band, C. Brainerd, R. Breedon, D. Burns, M. Calderon De La Barca Sanchez, M. Chertok, J. Conway, R. Conway, P. T. Cox, R. Erbacher, C. Flores, G. Funk, W. Ko, O. Kukral, R. Lander, C. Mclean, M. Mulhearn, D. Pellett, J. Pilot, S. Shalhout, M. Shi, D. Stolp, D. Taylor, K. Tos, M. Tripathi, Z. Wang, M. Bachtis, C. Bravo, R. Cousins, A. Dasgupta, A. Florent, J. Hauser, M. Ignatenko, N. Mccoll, S. Regnard, D. Saltzberg, C. Schnaible, V. Valuev, E. Bouvier, K. Burt, R. Clare, J. W. Gary, S. M. A. Ghiasi Shirazi, G. Hanson, G. Karapostoli, E. Kennedy, F. Lacroix, O. R. Long, M. Olmedo Negrete, M. I. Paneva, W. Si, L. Wang, H. Wei, S. Wimpenny, B. R. Yates, J. G. Branson, S. Cittolin, M. Derdzinski, R. Gerosa, D. Gilbert, B. Hashemi, A. Holzner, D. Klein, G. Kole, V. Krutelyov, J. Letts, M. Masciovecchio, D. Olivito, S. Padhi, M. Pieri, M. Sani, V. Sharma, S. Simon, M. Tadel, A. Vartak, S. Wasserbaech, J. Wood, F. Würthwein, A. Yagil, G. Zevi Della Porta, N. Amin, R. Bhandari, J. Bradmiller-Feld, C. Campagnari, M. Citron, A. Dishaw, V. Dutta, M. Franco Sevilla, L. Gouskos, R. Heller, J. Incandela, A. Ovcharova, H. Qu, J. Richman, D. Stuart, I. Suarez, S. Wang, J. Yoo, D. Anderson, A. Bornheim, J. M. Lawhorn, H. B. Newman, T. Q. Nguyen, M. Spiropulu, J. R. Vlimant, R. Wilkinson, S. Xie, Z. Zhang, R. Y. Zhu, M. B. Andrews, T. Ferguson, T. Mudholkar, M. Paulini, M. Sun, I. Vorobiev, M. Weinberg, J. P. Cumalat, W. T. Ford, F. Jensen, A. Johnson, M. Krohn, S. Leontsinis, E. MacDonald, T. Mulholland, K. Stenson, K. A. Ulmer, S. R. Wagner, J. Alexander, J. Chaves, Y. Cheng, J. Chu, A. Datta, K. Mcdermott, N. Mirman, J. R. Patterson, D. Quach, A. Rinkevicius, A. Ryd, L. Skinnari, L. Soffi, S. M. Tan, Z. Tao, J. Thom, J. Tucker, P. Wittich, M. Zientek, S. Abdullin, M. Albrow, M. Alyari, G. Apollinari, A. Apresyan, A. Apyan, S. Banerjee, L. A. T. Bauerdick, A. Beretvas, J. Berryhill, P. C. Bhat, G. Bolla, K. Burkett, J. N. Butler, A. Canepa, G. B. Cerati, H. W. K. Cheung, F. Chlebana, M. Cremonesi, J. Duarte, V. D. Elvira, J. Freeman, Z. Gecse, E. Gottschalk, L. Gray, D. Green, S. Grünendahl, O. Gutsche, J. Hanlon, R. M. Harris, S. Hasegawa, J. Hirschauer, Z. Hu, B. Jayatilaka, S. Jindariani, M. Johnson, U. Joshi, B. Klima, M. J. Kortelainen, B. Kreis, S. Lammel, D. Lincoln, R. Lipton, M. Liu, T. Liu, J. Lykken, K. Maeshima, J. M. Marraffino, D. Mason, P. McBride, P. Merkel, S. Mrenna, S. Nahn, V. O’Dell, K. Pedro, O. Prokofyev, G. Rakness, L. Ristori, A. Savoy-Navarro, B. Schneider, E. Sexton-Kennedy, A. Soha, W. J. Spalding, L. Spiegel, S. Stoynev, J. Strait, N. Strobbe, L. Taylor, S. Tkaczyk, N. V. Tran, L. Uplegger, E. W. Vaandering, C. Vernieri, M. Verzocchi, R. Vidal, M. Wang, H. A. Weber, A. Whitbeck, D. Acosta, P. Avery, P. Bortignon, D. Bourilkov, A. Brinkerhoff, L. Cadamuro, A. Carnes, M. Carver, D. Curry, R. D. Field, S. V. Gleyzer, B. M. Joshi, J. Konigsberg, A. Korytov, P. Ma, K. Matchev, H. Mei, G. Mitselmakher, K. Shi, D. Sperka, J. Wang, S. Wang, Y. R. Joshi, S. Linn, A. Ackert, T. Adams, A. Askew, S. Hagopian, V. Hagopian, K. F. Johnson, T. Kolberg, G. Martinez, T. Perry, H. Prosper, A. Saha, A. Santra, V. Sharma, R. Yohay, M. M. Baarmand, V. Bhopatkar, S. Colafranceschi, M. Hohlmann, D. Noonan, M. Rahmani, T. Roy, F. Yumiceva, M. R. Adams, L. Apanasevich, D. Berry, R. R. Betts, R. Cavanaugh, X. Chen, S. Dittmer, O. Evdokimov, C. E. Gerber, D. A. Hangal, D. J. Hofman, K. Jung, J. Kamin, C. Mills, I. D. Sandoval Gonzalez, M. B. Tonjes, N. Varelas, H. Wang, X. Wang, Z. Wu, J. Zhang, M. Alhusseini, B. Bilki, W. Clarida, K. Dilsiz, S. Durgut, R. P. Gandrajula, M. Haytmyradov, V. Khristenko, J.-P. Merlo, A. Mestvirishvili, A. Moeller, J. Nachtman, H. Ogul, Y. Onel, F. Ozok, A. Penzo, C. Snyder, E. Tiras, J. Wetzel, B. Blumenfeld, A. Cocoros, N. Eminizer, D. Fehling, L. Feng, A. V. Gritsan, W. T. Hung, P. Maksimovic, J. Roskes, U. Sarica, M. Swartz, M. Xiao, C. You, A. Al-bataineh, P. Baringer, A. Bean, S. Boren, J. Bowen, A. Bylinkin, J. Castle, S. Khalil, A. Kropivnitskaya, D. Majumder, W. Mcbrayer, M. Murray, C. Rogan, S. Sanders, E. Schmitz, J. D. Tapia Takaki, Q. Wang, A. Ivanov, K. Kaadze, D. Kim, Y. Maravin, D. R. Mendis, T. Mitchell, A. Modak, A. Mohammadi, L. K. Saini, N. Skhirtladze, F. Rebassoo, D. Wright, A. Baden, O. Baron, A. Belloni, S. C. Eno, Y. Feng, C. Ferraioli, N. J. Hadley, S. Jabeen, G. Y. Jeng, R. G. Kellogg, J. Kunkle, A. C. Mignerey, F. Ricci-Tam, Y. H. Shin, A. Skuja, S. C. Tonwar, K. Wong, D. Abercrombie, B. Allen, V. Azzolini, A. Baty, G. Bauer, R. Bi, S. Brandt, W. Busza, I. A. Cali, M. D’Alfonso, Z. Demiragli, G. Gomez Ceballos, M. Goncharov, P. Harris, D. Hsu, M. Hu, Y. Iiyama, G. M. Innocenti, M. Klute, D. Kovalskyi, Y.-J. Lee, P. D. Luckey, B. Maier, A. C. Marini, C. Mcginn, C. Mironov, S. Narayanan, X. Niu, C. Paus, C. Roland, G. Roland, G. S. F. Stephans, K. Sumorok, K. Tatar, D. Velicanu, J. Wang, T. W. Wang, B. Wyslouch, S. Zhaozhong, A. C. Benvenuti, R. M. Chatterjee, A. Evans, P. Hansen, S. Kalafut, Y. Kubota, Z. Lesko, J. Mans, S. Nourbakhsh, N. Ruckstuhl, R. Rusack, J. Turkewitz, M. A. Wadud, J. G. Acosta, S. Oliveros, E. Avdeeva, K. Bloom, D. R. Claes, C. Fangmeier, F. Golf, R. Gonzalez Suarez, R. Kamalieddin, I. Kravchenko, J. Monroy, J. E. Siado, G. R. Snow, B. Stieger, A. Godshalk, C. Harrington, I. Iashvili, A. Kharchilava, D. Nguyen, A. Parker, S. Rappoccio, B. Roozbahani, G. Alverson, E. Barberis, C. Freer, A. Hortiangtham, D. M. Morse, T. Orimoto, R. Teixeira De Lima, T. Wamorkar, B. Wang, A. Wisecarver, D. Wood, S. Bhattacharya, O. Charaf, K. A. Hahn, N. Mucia, N. Odell, M. H. Schmitt, K. Sung, M. Trovato, M. Velasco, R. Bucci, N. Dev, M. Hildreth, K. Hurtado Anampa, C. Jessop, D. J. Karmgard, N. Kellams, K. Lannon, W. Li, N. Loukas, N. Marinelli, F. Meng, C. Mueller, Y. Musienko, M. Planer, A. Reinsvold, R. Ruchti, P. Siddireddy, G. Smith, S. Taroni, M. Wayne, A. Wightman, M. Wolf, A. Woodard, J. Alimena, L. Antonelli, B. Bylsma, L. S. Durkin, S. Flowers, B. Francis, A. Hart, C. Hill, W. Ji, T. Y. Ling, W. Luo, B. L. Winer, H. W. Wulsin, S. Cooperstein, P. Elmer, J. Hardenbrook, P. Hebda, S. Higginbotham, A. Kalogeropoulos, D. Lange, M. T. Lucchini, J. Luo, D. Marlow, K. Mei, I. Ojalvo, J. Olsen, C. Palmer, P. Piroué, J. Salfeld-Nebgen, D. Stickland, C. Tully, S. Malik, S. Norberg, A. Barker, V. E. Barnes, L. Gutay, M. Jones, A. W. Jung, A. Khatiwada, B. Mahakud, D. H. Miller, N. Neumeister, C. C. Peng, H. Qiu, J. F. Schulte, J. Sun, F. Wang, R. Xiao, W. Xie, T. Cheng, J. Dolen, N. Parashar, Z. Chen, K. M. Ecklund, S. Freed, F. J. M. Geurts, M. Kilpatrick, W. Li, B. Michlin, B. P. Padley, J. Roberts, J. Rorie, W. Shi, Z. Tu, J. Zabel, A. Zhang, A. Bodek, P. de Barbaro, R. Demina, Y. t. Duh, J. L. Dulemba, C. Fallon, T. Ferbel, M. Galanti, A. Garcia-Bellido, J. Han, O. Hindrichs, A. Khukhunaishvili, K. H. Lo, P. Tan, R. Taus, M. Verzetti, A. Agapitos, J. P. Chou, Y. Gershtein, T. A. Gómez Espinosa, E. Halkiadakis, M. Heindl, E. Hughes, S. Kaplan, R. Kunnawalkam Elayavalli, S. Kyriacou, A. Lath, R. Montalvo, K. Nash, M. Osherson, H. Saka, S. Salur, S. Schnetzer, D. Sheffield, S. Somalwar, R. Stone, S. Thomas, P. Thomassen, M. Walker, A. G. Delannoy, J. Heideman, G. Riley, K. Rose, S. Spanier, K. Thapa, O. Bouhali, A. Castaneda Hernandez, A. Celik, M. Dalchenko, M. De Mattia, A. Delgado, S. Dildick, R. Eusebi, J. Gilmore, T. Huang, T. Kamon, S. Luo, R. Mueller, Y. Pakhotin, R. Patel, A. Perloff, L. Perniè, D. Rathjens, A. Safonov, A. Tatarinov, N. Akchurin, J. Damgov, F. De Guio, P. R. Dudero, S. Kunori, K. Lamichhane, S. W. Lee, T. Mengke, S. Muthumuni, T. Peltola, S. Undleeb, I. Volobouev, Z. Wang, S. Greene, A. Gurrola, R. Janjam, W. Johns, C. Maguire, A. Melo, H. Ni, K. Padeken, J. D. Ruiz Alvarez, P. Sheldon, S. Tuo, J. Velkovska, M. Verweij, Q. Xu, M. W. Arenton, P. Barria, B. Cox, R. Hirosky, M. Joyce, A. Ledovskoy, H. Li, C. Neu, T. Sinthuprasith, Y. Wang, E. Wolfe, F. Xia, R. Harr, P. E. Karchin, N. Poudyal, J. Sturdy, P. Thapa, S. Zaleski, M. Brodski, J. Buchanan, C. Caillol, D. Carlsmith, S. Dasu, L. Dodd, S. Duric, B. Gomber, M. Grothe, M. Herndon, A. Hervé, U. Hussain, P. Klabbers, A. Lanaro, A. Levine, K. Long, R. Loveless, T. Ruggles, A. Savin, N. Smith, W. H. Smith, N. Woods, CMS Collaboration

**Affiliations:** 1 Yerevan Physics Institute, Yerevan, Armenia; 2 Institut für Hochenergiephysik, Vienna, Austria; 3 Institute for Nuclear Problems, Minsk, Belarus; 4 Universiteit Antwerpen, Antwerp, Belgium; 5 Vrije Universiteit Brussel, Brussels, Belgium; 6 Université Libre de Bruxelles, Brussels, Belgium; 7 Ghent University, Ghent, Belgium; 8 Université Catholique de Louvain, Louvain-la-Neuve, Belgium; 9 Centro Brasileiro de Pesquisas Fisicas, Rio de Janeiro, Brazil; 10 Universidade do Estado do Rio de Janeiro, Rio de Janeiro, Brazil; 11 Universidade Estadual Paulista, Universidade Federal do ABC, São Paulo, Brazil; 12 Institute for Nuclear Research and Nuclear Energy, Bulgarian Academy of Sciences, Sofia, Bulgaria; 13 University of Sofia, Sofia, Bulgaria; 14 Beihang University, Beijing, China; 15 Institute of High Energy Physics, Beijing, China; 16 State Key Laboratory of Nuclear Physics and Technology, Peking University, Beijing, China; 17 Tsinghua University, Beijing, China; 18 Universidad de Los Andes, Bogota, Colombia; 19 University of Split, Faculty of Electrical Engineering, Mechanical Engineering and Naval Architecture, Split, Croatia; 20 University of Split, Faculty of Science, Split, Croatia; 21 Institute Rudjer Boskovic, Zagreb, Croatia; 22 University of Cyprus, Nicosia, Cyprus; 23 Charles University, Prague, Czech Republic; 24 Escuela Politecnica Nacional, Quito, Ecuador; 25 Universidad San Francisco de Quito, Quito, Ecuador; 26 Academy of Scientific Research and Technology of the Arab Republic of Egypt, Egyptian Network of High Energy Physics, Cairo, Egypt; 27 National Institute of Chemical Physics and Biophysics, Tallinn, Estonia; 28 Department of Physics, University of Helsinki, Helsinki, Finland; 29 Helsinki Institute of Physics, Helsinki, Finland; 30 Lappeenranta University of Technology, Lappeenranta, Finland; 31 IRFU, CEA, Université Paris-Saclay, Gif-sur-Yvette, France; 32 Laboratoire Leprince-Ringuet, Ecole polytechnique, CNRS/IN2P3, Université Paris-Saclay, Palaiseau, France; 33 Université de Strasbourg, CNRS, IPHC UMR 7178, Strasbourg, France; 34 Centre de Calcul de l’Institut National de Physique Nucleaire et de Physique des Particules, CNRS/IN2P3, Villeurbanne, France; 35 Université de Lyon, Université Claude Bernard Lyon 1, CNRS-IN2P3, Institut de Physique Nucléaire de Lyon, Villeurbanne, France; 36 Georgian Technical University, Tbilisi, Georgia; 37 Tbilisi State University, Tbilisi, Georgia; 38 RWTH Aachen University, I. Physikalisches Institut, Aachen, Germany; 39 RWTH Aachen University, III. Physikalisches Institut A, Aachen, Germany; 40 RWTH Aachen University, III. Physikalisches Institut B, Aachen, Germany; 41 Deutsches Elektronen-Synchrotron, Hamburg, Germany; 42 University of Hamburg, Hamburg, Germany; 43 Karlsruher Institut fuer Technologie, Karlsruhe, Germany; 44 Institute of Nuclear and Particle Physics (INPP), NCSR Demokritos, Aghia Paraskevi, Greece; 45 National and Kapodistrian University of Athens, Athens, Greece; 46 National Technical University of Athens, Athens, Greece; 47 University of Ioánnina, Ioánnina, Greece; 48 MTA-ELTE Lendület CMS Particle and Nuclear Physics Group, Eötvös Loránd University, Budapest, Hungary; 49 Wigner Research Centre for Physics, Budapest, Hungary; 50 Institute of Nuclear Research ATOMKI, Debrecen, Hungary; 51 Institute of Physics, University of Debrecen, Debrecen, Hungary; 52 Indian Institute of Science (IISc), Bangalore, India; 53 National Institute of Science Education and Research, HBNI, Bhubaneswar, India; 54 Panjab University, Chandigarh, India; 55 University of Delhi, Delhi, India; 56 Saha Institute of Nuclear Physics, HBNI, Kolkata, India; 57 Indian Institute of Technology Madras, Madras, India; 58 Bhabha Atomic Research Centre, Mumbai, India; 59 Tata Institute of Fundamental Research-A, Mumbai, India; 60 Tata Institute of Fundamental Research-B, Mumbai, India; 61 Indian Institute of Science Education and Research (IISER), Pune, India; 62 Institute for Research in Fundamental Sciences (IPM), Tehran, Iran; 63 University College Dublin, Dublin, Ireland; 64 INFN Sezione di Bari, Università di Bari, Politecnico di Bari, Bari, Italy; 65 INFN Sezione di Bologna, Università di Bologna, Bologna, Italy; 66 INFN Sezione di Catania, Università di Catania, Catania, Italy; 67 INFN Sezione di Firenze, Università di Firenze, Florence, Italy; 68 INFN Laboratori Nazionali di Frascati, Frascati, Italy; 69 INFN Sezione di Genova, Università di Genova, Genova, Italy; 70 INFN Sezione di Milano-Bicocca, Università di Milano-Bicocca, Milan, Italy; 71 INFN Sezione di Napoli, Università di Napoli ’Federico II’ , Napoli, Italy, Università della Basilicata, Potenza, Italy, Università G. Marconi, Rome, Italy; 72 INFN Sezione di Padova, Università di Padova, Padova, Italy, Università di Trento, Trento, Italy; 73 INFN Sezione di Pavia, Università di Pavia, Pavia, Italy; 74 INFN Sezione di Perugia, Università di Perugia, Perugia, Italy; 75 INFN Sezione di Pisa, Università di Pisa, Scuola Normale Superiore di Pisa, Pisa, Italy; 76 INFN Sezione di Roma, Sapienza Università di Roma, Rome, Italy; 77 INFN Sezione di Torino, Università di Torino, Torino, Italy, Università del Piemonte Orientale, Novara, Italy; 78 INFN Sezione di Trieste, Università di Trieste, Trieste, Italy; 79 Kyungpook National University, Daegu, Korea; 80 Chonnam National University, Institute for Universe and Elementary Particles, Kwangju, Korea; 81 Hanyang University, Seoul, Korea; 82 Korea University, Seoul, Korea; 83 Sejong University, Seoul, Korea; 84 Seoul National University, Seoul, Korea; 85 University of Seoul, Seoul, Korea; 86 Sungkyunkwan University, Suwon, Korea; 87 Vilnius University, Vilnius, Lithuania; 88 National Centre for Particle Physics, Universiti Malaya, Kuala Lumpur, Malaysia; 89 Centro de Investigacion y de Estudios Avanzados del IPN, Mexico City, Mexico; 90 Universidad Iberoamericana, Mexico City, Mexico; 91 Benemerita Universidad Autonoma de Puebla, Puebla, Mexico; 92 Universidad Autónoma de San Luis Potosí, San Luis Potosí, Mexico; 93 University of Auckland, Auckland, New Zealand; 94 University of Canterbury, Christchurch, New Zealand; 95 National Centre for Physics, Quaid-I-Azam University, Islamabad, Pakistan; 96 National Centre for Nuclear Research, Swierk, Poland; 97 Institute of Experimental Physics, Faculty of Physics, University of Warsaw, Warsaw, Poland; 98 Laboratório de Instrumentação e Física Experimental de Partículas, Lisbon, Portugal; 99 Joint Institute for Nuclear Research, Dubna, Russia; 100 Petersburg Nuclear Physics Institute, Gatchina (St. Petersburg), Russia; 101 Institute for Nuclear Research, Moscow, Russia; 102 Institute for Theoretical and Experimental Physics, Moscow, Russia; 103 Moscow Institute of Physics and Technology, Moscow, Russia; 104 National Research Nuclear University ‘Moscow Engineering Physics Institute’ (MEPhI), Moscow, Russia; 105 P.N. Lebedev Physical Institute, Moscow, Russia; 106 Skobeltsyn Institute of Nuclear Physics, Lomonosov Moscow State University, Moscow, Russia; 107 Novosibirsk State University (NSU), Novosibirsk, Russia; 108 Institute for High Energy Physics of National Research Centre ’Kurchatov Institute’, Protvino, Russia; 109 National Research Tomsk Polytechnic University, Tomsk, Russia; 110 University of Belgrade, Faculty of Physics and Vinca Institute of Nuclear Sciences, Belgrade, Serbia; 111 Centro de Investigaciones Energéticas Medioambientales y Tecnológicas (CIEMAT), Madrid, Spain; 112 Universidad Autónoma de Madrid, Madrid, Spain; 113 Universidad de Oviedo, Oviedo, Spain; 114 Instituto de Física de Cantabria (IFCA), CSIC-Universidad de Cantabria, Santander, Spain; 115 CERN, European Organization for Nuclear Research, Geneva, Switzerland; 116 Paul Scherrer Institut, Villigen, Switzerland; 117 ETH Zurich-Institute for Particle Physics and Astrophysics (IPA), Zurich, Switzerland; 118 Universität Zürich, Zurich, Switzerland; 119 National Central University, Chung-Li, Taiwan; 120 National Taiwan University (NTU), Taipei, Taiwan; 121 Department of Physics, Faculty of Science, Chulalongkorn University, Bangkok, Thailand; 122 Physics Department, Science and Art Faculty, Çukurova University, Adana, Turkey; 123 Middle East Technical University, Physics Department, Ankara, Turkey; 124 Bogazici University, Istanbul, Turkey; 125 Istanbul Technical University, Istanbul, Turkey; 126 Institute for Scintillation Materials of National Academy of Science of Ukraine, Kharkov, Ukraine; 127 National Scientific Center, Kharkov Institute of Physics and Technology, Kharkov, Ukraine; 128 University of Bristol, Bristol, UK; 129 Rutherford Appleton Laboratory, Didcot, UK; 130 Imperial College, London, UK; 131 Brunel University, Uxbridge, UK; 132 Baylor University, Waco, USA; 133 Catholic University of America, Washington DC, USA; 134 The University of Alabama, Tuscaloosa, USA; 135 Boston University, Boston, USA; 136 Brown University, Providence, USA; 137 University of California, Davis, Davis, USA; 138 University of California, Los Angeles, USA; 139 University of California, Riverside, Riverside, USA; 140 University of California, San Diego, La Jolla, USA; 141 Department of Physics, University of California, Santa Barbara, Santa Barbara, USA; 142 California Institute of Technology, Pasadena, USA; 143 Carnegie Mellon University, Pittsburgh, USA; 144 University of Colorado Boulder, Boulder, USA; 145 Cornell University, Ithaca, USA; 146 Fermi National Accelerator Laboratory, Batavia, USA; 147 University of Florida, Gainesville, USA; 148 Florida International University, Miami, USA; 149 Florida State University, Tallahassee, USA; 150 Florida Institute of Technology, Melbourne, USA; 151 University of Illinois at Chicago (UIC), Chicago, USA; 152 The University of Iowa, Iowa City, USA; 153 Johns Hopkins University, Baltimore, USA; 154 The University of Kansas, Lawrence, USA; 155 Kansas State University, Manhattan, USA; 156 Lawrence Livermore National Laboratory, Livermore, USA; 157 University of Maryland, College Park, USA; 158 Massachusetts Institute of Technology, Cambridge, USA; 159 University of Minnesota, Minneapolis, USA; 160 University of Mississippi, Oxford, USA; 161 University of Nebraska-Lincoln, Lincoln, USA; 162 State University of New York at Buffalo, Buffalo, USA; 163 Northeastern University, Boston, USA; 164 Northwestern University, Evanston, USA; 165 University of Notre Dame, Notre Dame, USA; 166 The Ohio State University, Columbus, USA; 167 Princeton University, Princeton, USA; 168 University of Puerto Rico, Mayaguez, USA; 169 Purdue University, West Lafayette, USA; 170 Purdue University Northwest, Hammond, USA; 171 Rice University, Houston, USA; 172 University of Rochester, Rochester, USA; 173 Rutgers, The State University of New Jersey, Piscataway, USA; 174 University of Tennessee, Knoxville, USA; 175 Texas A & M University, College Station, USA; 176 Texas Tech University, Lubbock, USA; 177 Vanderbilt University, Nashville, USA; 178 University of Virginia, Charlottesville, USA; 179 Wayne State University, Detroit, USA; 180 University of Wisconsin-Madison, Madison, WI USA

**Keywords:** CMS, Physics, QCD, Jets, Azimuthal angle, MC generators

## Abstract

A measurement for inclusive 2- and 3-jet events of the azimuthal correlation between the two jets with the largest transverse momenta, $\varDelta \phi _{12}$, is presented. The measurement considers events where the two leading jets are nearly collinear (“back-to-back”) in the transverse plane and is performed for several ranges of the leading jet transverse momentum. Proton-proton collision data collected with the CMS experiment at a center-of-mass energy of $13\,\text {Te}\text {V}$ and corresponding to an integrated luminosity of $35.9{\,\text {fb}^{-1}}$ are used. Predictions based on calculations using matrix elements at leading-order and next-to-leading-order accuracy in perturbative quantum chromodynamics supplemented with leading-log parton showers and hadronization are generally in agreement with the measurements. Discrepancies between the measurement and theoretical predictions are as large as 15%, mainly in the region $177^\circ< \varDelta \phi _{12} < 180^\circ$. The 2- and 3-jet measurements are not simultaneously described by any of models.

## Introduction

Collimated streams of particles (jets) can be produced in highly energetic parton-parton interactions in proton-proton (s) collisions, and their properties are described by the theory of strong interactions, quantum chromodynamics (QCD). In the lowest order perturbative QCD (pQCD), two jets with high transverse momenta $p_{{\mathrm{T}}}$ are produced “back-to-back” in the transverse plane. Higher order corrections lead to deviations from this configuration. Experimentally, this can be investigated by the measurement of the azimuthal separation, $\varDelta \phi _{12} =|\phi _\text {jet1}-\phi _\text {jet2} |$, between the two leading $p_{{\mathrm{T}}}$ jets in the transverse plane. Within the framework of pQCD, a final state with three or more partons is required for significant deviations from $\varDelta \phi _{12} =180^\circ$. However, when deviations of $\varDelta \phi _{12}$ from $180^\circ$ are small, a pQCD calculation at a fixed order in the strong coupling $\alpha _S$ becomes unstable and a resummation of soft parton emissions to all orders in $\alpha _S$ has to be performed. This resummation is approximated through the use of parton showers in Monte Carlo (MC) event generators.

Azimuthal correlations in inclusive 2-jet events have been measured previously by the D0 Collaboration in ${\text {p}} {\bar{\mathrm{p}}}$ collisions at a center-of-mass energy of $\sqrt{s}=1.96\,\text {Te}\text {V}$ [1, 2], in ${\text {p}} {\text {p}}$ collisions by the ATLAS Collaboration at $\sqrt{s}=7\,\text {Te}\text {V}$ [3], and by the CMS Collaboration at $\sqrt{s}=7$, 8, and $13\,\text {Te}\text {V}$ [4–6], but none of the measurements considered in detail the region close to the back-to-back configuration. A detailed study of azimuthal correlations close to the back-to-back configuration allows a more precise test of different resummation strategies, and it is a first step towards an improved understanding of the effects of soft initial and final state gluons [7, 8]. The leading- and next-to-leading-logarithm contributions to the dijet azimuthal angular correlation have been investigated in [9–11]. The effects of applying a transverse momentum dependent parton showering to the dijet azimuthal angular correlation were studied in [12].

In this article measurements are reported of the normalized inclusive 2-jet distribution as a function of the azimuthal separation $\varDelta \phi _{12}$ between the two leading $p_{{\mathrm{T}}}$ jets (jets 1 and 2),

1 $$\begin{aligned} \frac{1}{\sigma _{p_{{\mathrm{T}}} ^{\mathrm{max}}}} \frac{d\sigma }{d\varDelta \phi _{12}}, \end{aligned}$$

in several intervals of the leading jet $p_{{\mathrm{T}}}$ ($p_{{\mathrm{T}}} ^{\mathrm{max}}$) within the rapidity range $|y |<2.5$. The total dijet cross section $\sigma _{p_{{\mathrm{T}}} ^{\mathrm{max}}}$ is measured within each range of $p_{{\mathrm{T}}} ^{\mathrm{max}}$ integrated over the full range in $\varDelta \phi _{12}$. The binning of the measurement presented here is much finer than that of Ref. [6]. We consider $\varDelta \phi _{12}$ in the range $170^{\circ } < \varDelta \phi _{12} \le 180^{\circ }$.

The inclusive 3-jet distributions, differential in $\varDelta \phi _{12}$ and $p_{{\mathrm{T}}} ^{\mathrm{max}}$, with the $p_{{\mathrm{T}}}$ of third highest $p_{{\mathrm{T}}}$ jet typically being 1-2 orders of magnitude smaller than $p_{{\mathrm{T}}} ^{\mathrm{max}}$, are also suitable to test resummation effects arising from the presence of multiple scales in the interaction. Measurements of the inclusive 3-jet distribution normalized to $\sigma _{p_{{\mathrm{T}}} ^{\mathrm{max}}}$ are also presented, for several ranges of $p_{{\mathrm{T}}} ^{\mathrm{max}}$, and within $|y |<2.5$.

The measurements are performed using data collected from p p collisions at $\sqrt{s}=13\,\text {Te}\text {V}$ during 2016 with the CMS experiment at the CERN LHC, corresponding to an integrated luminosity of 35.9$\,\text {fb}^{-1}$.

## The CMS detector

The central feature of the CMS detector is a superconducting solenoid, 13 m in length and 6 m in inner diameter, providing an axial magnetic field of 3.8 T. Within the solenoid volume are a silicon pixel and strip tracker, a lead tungstate crystal electromagnetic calorimeter (ECAL) and a brass and scintillator hadron calorimeter (HCAL), each composed of a barrel and two endcap sections. Charged-particle trajectories are measured by the tracker with full azimuthal coverage within pseudorapidities $|\eta |<2.5$. The ECAL, which is equipped with a preshower detector in the endcaps, and the HCAL cover the region $|\eta |<3.0$. Forward calorimeters extend the pseudorapidity coverage provided by the barrel and endcap detectors to the region $3.0<|\eta |<5.2$. Finally, muons are measured up to $|\eta |<2.4$ by gas-ionization detectors embedded in the steel flux-return yoke outside the solenoid. A detailed description of the CMS detector together with a definition of the coordinate system used and the relevant kinematic variables can be found in Ref. [13].

## Theoretical predictions

Simulations from leading-order (LO) and next-to-LO (NLO) MC event generators are investigated. Among the LO event generators, both pythia 8 [14] (version 8.219) and herwig++ [15] (version 2.7.1) are used for predictions because they feature different parton showering (PS) algorithms for soft and collinear parton radiation at leading-log accuracy. In pythia 8 the PS emissions cover a region of phase space ordered in *x* (fraction of the proton momentum carried by the parton) and the $p_{{\mathrm{T}}}$ of the emitted parton, whereas in herwig++ the parton emissions are ordered in *x* and the angle of the radiated parton (angular ordering). The Lund string model [16] is used for hadronization in pythia 8  [14], whereas in herwig++ the cluster fragmentation model [17] is applied. Multiparton interactions (MPI) are simulated in pythia 8 (tune CUETP8M1 [18] with the parton distribution function (PDF) set NNPDF2.3LO [19, 20]) and in herwig++ (tune CUETHppS1 [18] with the PDF set CTEQ6L1 [21]) with parameters tuned to measurements in p p collisions at the LHC and ${\text {p}} {\bar{\mathrm{p}}}$ collisions at the Tevatron.

The ${\textsc {MadGraph}} {}5\_\text {a}{\textsc {mc@nlo}}$ [22] version 2.3.3 event generator (labelled as MadGraph in the following) interfaced with pythia 8 with tune CUETP8M1 is also used in the analysis. Processes with up to 4 final-state partons at LO accuracy are calculated using the NNPDF2.3LO PDF set. The $k_{{\mathrm{T}}}$-MLM matching procedure [23] is used with a matching scale of 10$\,\text {Ge}\text {V}$.

Among the NLO event generators, predictions obtained using the powheg box library [24–26] (version 2) with the PDF set NNPDF3.0NLO [27] are considered. The event generators pythia 8 (tune CUETP8M1) and herwig++ (tune CUETHppS1) are used to simulate PS, hadronization, and MPI. The powheg generator in dijet mode [28], referred to as ph-2j, provides an NLO dijet calculation, which is accurate to LO for the azimuthal correlation between the leading jets. The powheg generator in three-jet mode [29] (using the MiNLO scheme [30, 31]), referred to as ph-3j, provides an NLO $2\rightarrow 3$ calculation. For the ph-2j matrix elements (ME), a minimum $p_{{\mathrm{T}}}$ of 100 GeV is required on the partons in the Born process, while for the ph-3j ME the minimum is lowered to 10 GeV to ensure coverage of the full phase space. These thresholds are applied to optimize the generation of events in the phase space of interest. The matching between the powheg matrix element calculations and the pythia 8 underlying event (UE) [18] simulation is performed by using the shower-veto procedure (UserHook option 2 [14]). The matching between the powheg matrix element calculations and the herwig++ UE [18] is performed by using a truncated shower [24].

Events generated by pythia 8 (tune CUETP8M1), herwig++ (tune CUETHppS1), and MadGraph interfaced with pythia 8 (tune CUETP8M1) are passed through a full detector simulation based on Geant4 [32]. The simulated events events are reconstructed with standard CMS programs.

Table 1 summarizes the theoretical predictions used in the present analysis.

**Table 1:** Monte Carlo event generators, parton densities, and underlying event tunes used for comparison with measurements

Matrix element generator	Simulated diagrams	PDF set	Tune
pythia 8.219 [14]	2$\rightarrow$2 (LO)	NNPDF2.3LO [19, 20]	CUETP8M1 [18]
herwig++ 2.7.1 [15]	2$\rightarrow$2 (LO)	CTEQ6L1 [21]	CUETHppS1 [18]
MadGraph [22, 23]+ pythia 8.219 [14]	2$\rightarrow$2, 2$\rightarrow$3, 2$\rightarrow$4 (LO)	NNPDF2.3LO [19, 20]	CUETP8M1 [18]
ph-2j [24–26] + pythia 8.219 [14]	2$\rightarrow$2 (NLO)	NNPDF3.0NLO [27]	CUETP8M1 [18]
ph-2j [24–26] + herwig++ 2.7.1 [15]	2$\rightarrow$2 (NLO)	NNPDF3.0NLO [27]	CUETHppS1 [18]
ph-3j [24–26] + pythia 8.219 [14]	2$\rightarrow$3 (NLO)	NNPDF3.0NLO [27]	CUETP8M1 [18]

## Jet reconstruction and event selection

The measurements are based on data samples collected with single-jet high-level triggers [33, 34]. The five single-jet triggers require at least one jet in the event with $p_{{\mathrm{T}}} > 140$, 200, 320, 400, or $450 \,\text {Ge}\text {V}$ within the full rapidity coverage of the CMS calorimetry. Table 2 shows the various $p_{{\mathrm{T}}} ^{\mathrm{max}}$ regions accessed by the various triggers and the integrated luminosity for each trigger in the analysis. Each trigger is fully efficient for jets in the corresponding $p_{{\mathrm{T}}}$ range in Table 2.

**Table 2:** The integrated luminosity for each trigger sample in the analysis, and trigger used for each $p_{{\mathrm{T}}} ^{\mathrm{max}}$ range

HLT $p_{{\mathrm{T}}}$ threshold ($\text {Ge}\text {V}$ )	140	200	320	400	450
$\mathcal {L}$ ($\text {fb}^{-1}$)	0.024	0.11	1.77	5.2	36
$p_{{\mathrm{T}}} ^{\mathrm{max}}$ region ($\text {Ge}\text {V}$ )	200–300	300–400	400–500	500–600	>600

Particles are reconstructed and identified using a particle-flow (PF) algorithm [35], which utilizes an optimized combination of information from the various elements of the CMS detector. Jets are reconstructed by clustering the four-vectors of the PF candidates with the infrared- and collinear-safe anti-$k_{{\mathrm{T}}}$ clustering algorithm [36] with a distance parameter $R=0.4$. The clustering is performed with the FastJet package [37]. To reduce the contribution to the reconstructed jets from additional p p interactions within the same bunch crossing (pileup), the charged-hadron subtraction technique [38] is used to remove tracks identified as originating from pileup vertices. The average number of pileup interactions per single bunch crossing observed in the data is about 27. The pileup contribution from neutral hadrons is corrected using a jet-area-based correction technique [39].

For this analysis, jets with rapidity $|y |< 5.0$ are reconstructed. For both the inclusive 2- and 3-jet samples, the events are selected by requiring the two highest $p_{{\mathrm{T}}}$ jets to have $|y |< 2.5$ and $p_{{\mathrm{T}}} > 100\,\text {Ge}\text {V}$. For the inclusive 3-jet events a third jet with $p_{{\mathrm{T}}} >30\,\text {Ge}\text {V}$ and $|y |< 2.5$ is required. Contributions from pileup are negligible because the pileup removal algorithm has an efficiency of $\sim$99% for jets with $30<p_{{\mathrm{T}}} < 50\,\text {Ge}\text {V}$ and $|y |< 2.5$ [40].

## Measurements of the normalized inclusive 2- and 3-jet distributions

The normalized inclusive 2- and 3-jet distributions as a function of $\varDelta \phi _{12}$ are corrected for detector resolution. We achieve this by unfolding the observables to the level of stable final-state particles. In this way, a direct comparison of these measurements to results from other experiments and to QCD predictions is possible. Particles are considered stable if their mean decay length is larger than 1 cm.

The unfolding procedure is based on the D’Agostini algorithm [41], which is implemented in the RooUnfold package [42], by using a response matrix that maps the generated jets onto the jets reconstructed by the CMS detector. The regularization (number of iterations) of the unfolding procedure is chosen by comparing the difference in $\chi ^2$ between data and MC at detector level to that between data and MC at particle level. The consistency of the unfolding procedure is checked against the alternative TUnfold package [43, 44], which uses a least square minimization with Tikhonov regularization. Both methods provide equivalent results. The unfolding is performed in $\varDelta \phi _{12}$. The response matrices are obtained using simulated events from the pythia 8 event generator with the tune CUETP8M1. The difference between the unfolded distributions and the distributions at detector level range from $\sim$1% for the low $p_{{\mathrm{T}}} ^{\mathrm{max}}$ regions up to $\sim$5% for the high $p_{{\mathrm{T}}} ^{\mathrm{max}}$ regions.

The sources of systematic uncertainties arise primarily from the jet energy scale calibration (JES), the jet energy resolution (JER), the $\varDelta \phi _{12}$ resolution, and the model dependence of the unfolding matrix. The effect of migrations between $p_{{\mathrm{T}}} ^{\mathrm{max}}$ regions is very small because of the normalization of the cross sections in each $p_{{\mathrm{T}}} ^{\mathrm{max}}$ range and therefore is neglected.

The $\varDelta \phi _{12}$ resolution is ${\sim }0.5^{\circ }$, as obtained from fully simulated event samples from pythia 8 and MadGraph. A bin size of $1^{\circ }$ is a compromise between the ability to study the back-to-back region and the impact of the unfolding correction of $\sim$2%. In Ref. [6] the study is focused on a different $\varDelta \phi _{12}$ region, and a coarser bin size is chosen to account for the smaller size of the data sample.

Alternative response matrices are obtained by using the $\varDelta \phi _{12}$ resolution determined from fully simulated events. This resolution is varied by ±10%, an amount that is motivated by the observed difference between data and simulation. The resulting uncertainty is estimated to be below 1%.

An additional systematic uncertainty is caused by the dependence of the response matrix on the choice of the MC generator. Alternative response matrices are built using the herwig++ and MadGraph + pythia 8 event generators. Because this analysis uses a finer binning compared with that of Ref. [6], the sensitivity to the uncertainty in the unfolding is increased. The observed effect from bin migration is less than 2%.

The JER and shifts in the JES can cause events to migrate between the $p_{{\mathrm{T}}} ^{\mathrm{max}}$ regions. The JES uncertainties on the energy measurement are estimated to be 1–2% [38]. The resulting JES uncertainties in the normalized inclusive 2-jet distributions due to bin migrations are less than 2%, whereas for the normalized inclusive 3-jet distributions they are less than 3%. The effect of the JER uncertainties [38] is estimated by varying the JER parameters by one standard deviation up and down and comparing the results before and after the changes. The JER-induced uncertainties are less than 0.2% for the inclusive 2-jet $\varDelta \phi _{12}$ measurement and below 0.4% for the normalized inclusive 3-jet measurement.

## Comparison to theoretical predictions

In this section the measurements are compared with different theoretical predictions introduced in Section 3. In all figures displaying ratios, the solid band indicates the total experimental uncertainty and the error bars represent the statistical uncertainties from the simulation. In the figures displaying the normalized distributions, the error bars on the data represent the total experimental uncertainty and the error bars on the predictions represent the statistical uncertainty of the simulation. The uncertainties are often so small that the bars are not visible.

**Fig. 1:**
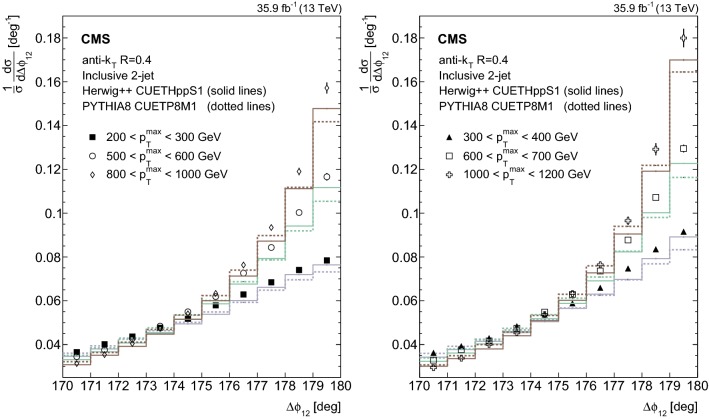
Normalized inclusive 2-jet distributions as a function of the azimuthal separation of the two leading jets $\varDelta \phi _{12}$ for different $p_{{\mathrm{T}}} ^{\mathrm{max}}$ regions. The data are represented by the markers and the theory by histograms. Overlaid with the data are predictions from the herwig++ event generator (solid lines) and pythia 8 (dotted lines). The total experimental uncertainty is depicted as error bars on the measurement

**Fig. 2:**
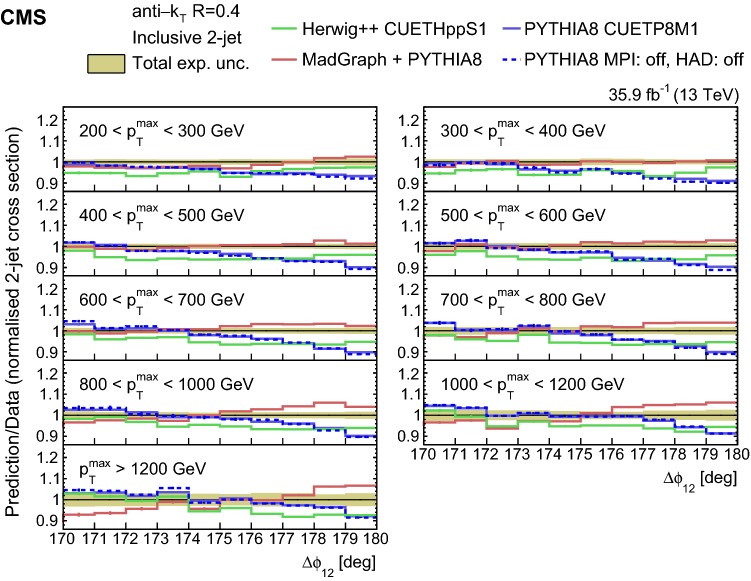
Ratios of the normalized inclusive 2-jet distributions for the pythia 8, herwig++, and MadGraph + pythia 8 predictions to data as a function of the azimuthal separation of the two leading jets $\varDelta \phi _{12}$, for all the $p_{{\mathrm{T}}} ^{\mathrm{max}}$ regions. The solid band indicates the total experimental uncertainty and the error bars on the MC points represent the statistical uncertainty of the simulated data

The unfolded normalized inclusive 2-jet distribution as a function of $\varDelta \phi _{12}$ is shown in Fig. 1, and compared with the predictions from herwig++ (solid lines ) and pythia 8 (dotted lines) for different $p_{{\mathrm{T}}} ^{\mathrm{max}}$ regions. The distributions are strongly peaked at $180^{\circ }$ and become steeper with increasing $p_{{\mathrm{T}}} ^{\mathrm{max}}$. The ratio of the pythia 8, herwig++, and MadGraph + pythia 8 event generator predictions to data are depicted in Fig. 2 for the inclusive 2-jet distributions in the nine $p_{{\mathrm{T}}} ^{\mathrm{max}}$ ranges. Among the event generators, pythia 8 and herwig++ show the largest deviations from the measurements for the $p_{{\mathrm{T}}} ^{\mathrm{max}} < 800$ GeV regions in the inclusive 2-jet case, and the MadGraph + pythia 8 event generator gives the best description in the same regions. The three generators show large deviations from the measurements in the $p_{{\mathrm{T}}} ^{\mathrm{max}} > 800$ GeV regions. The nonperturbative corrections are estimated to be small (below 1.5%) by comparing the predictions from pythia 8 without the simulation of multi-parton interactions and hadronization (dashed blue curve) to the predictions from pythia 8 when these effects are included (solid blue curve). The nonperturbative correction factors are available in HepData.

**Fig. 3:**
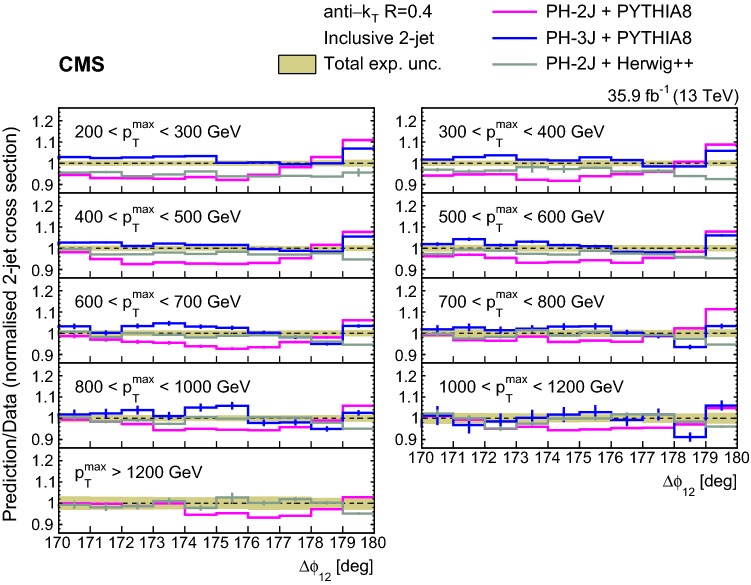
Ratios of the normalized inclusive 2-jet distributions for the ph-2j + pythia 8, ph-3j + pythia 8, and ph-2j + herwig++ predictions to data as a function of the azimuthal separation of the two leading jets $\varDelta \phi _{12}$, for all the $p_{{\mathrm{T}}} ^{\mathrm{max}}$ regions. The solid band indicates the total experimental uncertainty and the error bars on the MC points represent the statistical uncertainty of the simulated data. The ph-3j prediction is not shown for the highest bin in $p_{{\mathrm{T}}} ^{\mathrm{max}}$ because of the large statistical fluctuations

The ratios of the NLO predictions to data for the unfolded normalized inclusive 2-jet distributions for the different $p_{{\mathrm{T}}} ^{\mathrm{max}}$ regions are shown in Fig. 3. The NLO calculations considered are ph-2j + pythia 8, ph-2j + herwig++, and ph-3j + pythia 8. Among these NLO predictions ph-3j + pythia 8 agrees better with the data. The ph-2j + herwig++ prediction is similar to the one of ph-3j + pythia 8, except for the lowest $p_{{\mathrm{T}}} ^{\mathrm{max}}$ region.

**Fig. 4:**
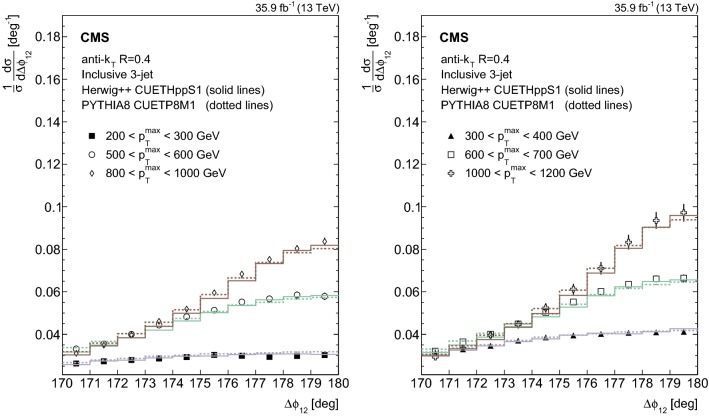
Normalized inclusive 3-jet distributions as a function of the azimuthal separation of the two leading jets $\varDelta \phi _{12}$ for different $p_{{\mathrm{T}}} ^{\mathrm{max}}$ regions. The data are represented by the markers and the theory by histograms. Overlaid with the data are predictions from the herwig++ event generator (solid lines) and pythia 8 (dotted lines). The total experimental uncertainty is depicted as error bars on the measurement

**Fig. 5:**
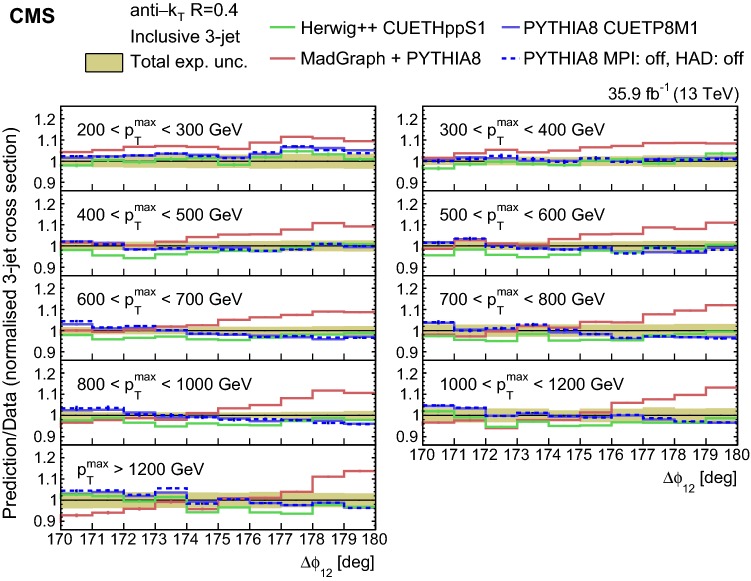
Ratios of the normalized inclusive 3-jet distributions for the pythia 8, herwig++, and MadGraph + pythia 8 predictions to data as a function of the azimuthal separation of the two leading jets $\varDelta \phi _{12}$, for all the $p_{{\mathrm{T}}} ^{\mathrm{max}}$ regions. The solid band indicates the total experimental uncertainty and the error bars on the MC points represent the statistical uncertainty of the simulated data

In Fig. 4 the unfolded normalized inclusive 3-jet distribution as a function of $\varDelta \phi _{12}$ are compared with the predictions from herwig++ (solid lines) and pythia 8 (dotted lines) for different $p_{{\mathrm{T}}} ^{\mathrm{max}}$ regions. The ratios of the normalized inclusive 3-jet distributions for the pythia 8, herwig++, and MadGraph + pythia 8 predictions to data are shown in Fig. 5 for the different $p_{{\mathrm{T}}} ^{\mathrm{max}}$ regions. In contrast to the 2-jet case, MadGraph + pythia 8 shows the largest deviations from the measurements close to $180^{\circ }$, whereas pythia 8 and herwig++ give a good description of the data.

**Fig. 6:**
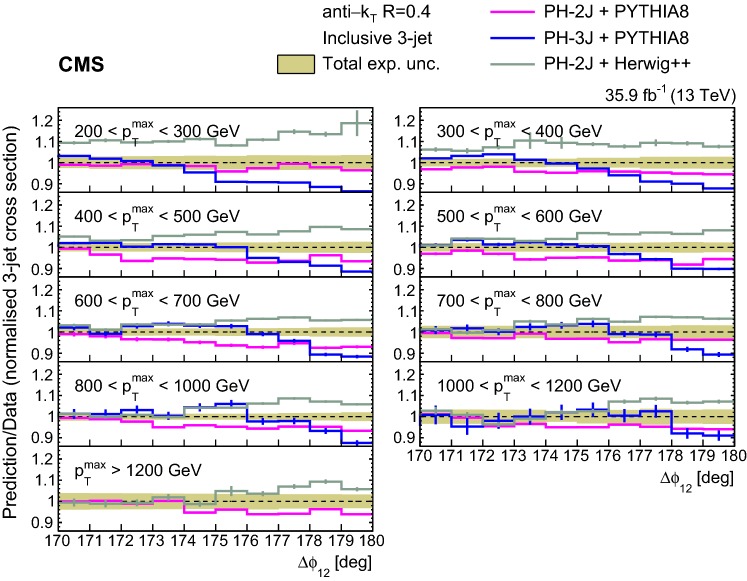
Ratios of the normalized inclusive 3-jet distributions for the ph-2j + pythia 8, ph-3j + pythia 8, and ph-2j + herwig++ predictions to data as a function of the azimuthal separation of the two leading jets $\varDelta \phi _{12}$, for all $p_{{\mathrm{T}}} ^{\mathrm{max}}$ regions. The solid band indicates the total experimental uncertainty and the error bars on the MC points represent the statistical uncertainty of the simulated data. The ph-3j prediction is not shown for the highest bin in $p_{{\mathrm{T}}} ^{\mathrm{max}}$ because of the large statistical fluctuations

The ratios of the NLO predictions from ph-2j + pythia 8, ph-2j + herwig++, and ph-3j + pythia 8 to data for the normalized inclusive 3-jet distributions are shown in Fig. 6. All the considered NLO+PS predictions fail to describe the measurements close to $180^\circ$. The predictions from ph-3j and MadGraph (Fig. 5) behave very differently, in contrast to their similar trend in the inclusive 2-jet case.

Since pythia 8, ph-2j + pythia 8, ph-3j + pythia 8, and MadGraph + pythia 8 use the same parton shower, the observed differences in the predictions can be attributed to the treatment of the additional partons present in the powheg and MadGraph ME.

In general we observe that the $\varDelta \phi _{12}$ region close to $180^\circ$ is not well described by the predictions. The predictions agree better with the measurements for increasing $p_{{\mathrm{T}}} ^{\mathrm{max}}$ and moving further away from the back-to-back region in $\varDelta \phi _{12}$, where the contribution of resummation effects becomes smaller [10]. The fact that none of the generators is able to describe the 2- and 3-jet measurements simultaneously suggests that the observed differences (of the order of 10%) are related to the way soft partons are simulated within the PS. The observed differences between $p_{{\mathrm{T}}}$ and angular ordered PS for the LO generators pythia 8 and herwig++ are small (Figs. 2, 5) compared to the MadGraph predictions, which can be attributed to the presence of higher order ME.

**Fig. 7:**
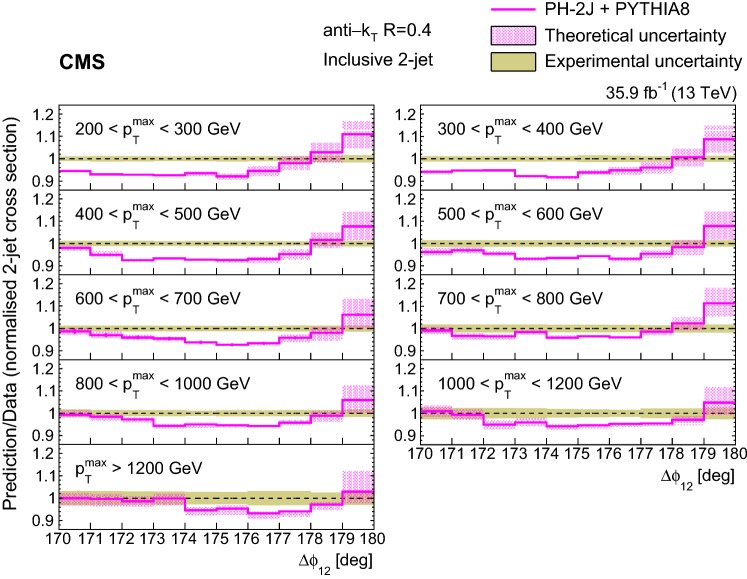
Ratios of the normalized inclusive 2-jet distributions for the ph-2j + pythia 8 predictions to data as a function of the azimuthal separation of the two leading jets $\varDelta \phi _{12}$, for all $p_{{\mathrm{T}}} ^{\mathrm{max}}$ regions. The solid beige band indicates the total experimental uncertainty and the hatched band represents the total theoretical uncertainty

**Fig. 8:**
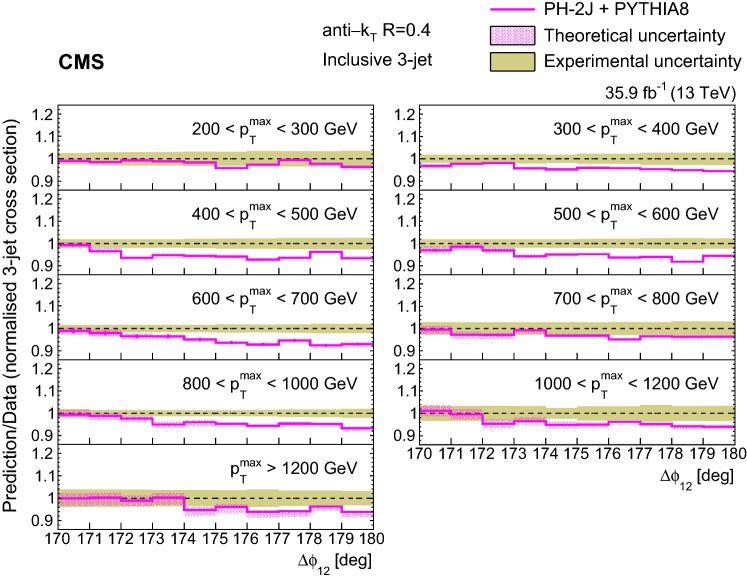
Ratios of the normalized inclusive 3-jet distributions for the ph-2j + pythia 8 predictions to data as a function of the azimuthal separation of the two leading jets $\varDelta \phi _{12}$, for all $p_{{\mathrm{T}}} ^{\mathrm{max}}$ regions. The solid beige band indicates the total experimental uncertainty, the hatched band represents the total theoretical uncertainty

The theoretical calculations have an intrinsic uncertainty arising from the freedom of choice of the renormalization and factorization scales ($\mu _r$ and $\mu _f$), the choice of the PDF and $\alpha _S (m_{{{Z}}})$, and the modeling of nonperturbative effects and PS. The total theoretical uncertainty is the quadratic sum of the uncertainties from the scale, PDF, $\alpha _S$, and PS variations. Despite the better agreement of ph-3j, the ph-2j event generator is used instead for the estimation of the scale, PDF, and $\alpha _S$ uncertainties, because of the larger event sample. For the estimation of the PS uncertainty pythia 8 is utilized. The following four sources of theoretical uncertainties are analyzed:

- The uncertainties due to the renormalization and factorization scales of the hard process are evaluated by varying the default choice $\mu _r = \mu _f = p_{{\mathrm{T}}}$ of the underlying Born configuration between $p_{{\mathrm{T}}}$/2 and 2$p_{{\mathrm{T}}}$. The envelope of the following seven combinations is considered: $(\mu _r/p_{{\mathrm{T}}}, \mu _f/p_{{\mathrm{T}}}) = (0.5, 0.5)$, (0.5, 1), (1, 0.5), (1, 1), (1, 2), (2, 1), and (2, 2).
- The PDF uncertainties are evaluated according to the prescriptions for the NNPDF3.0 NLO PDF set. There are 100 replicas of the NNPDF3.0 NLO PDF set. For each replica the cross section is calculated and the uncertainty is taken as the envelope from all the replicas.
- The uncertainty due to the value of the strong coupling $\alpha _S$ is obtained by a variation of $\alpha _S (m_{{{Z}}})$ by ±0.001, as recommended in Ref. [45].
- The uncertainty due to PS is evaluated with the pythia 8 event generator by varying the default renormalization scale choice $\mu _r = p_{{\mathrm{T}}}$ of the branching in initial state (ISR) and final state radiation (FSR) between $\mu _r/2$ and 2$\mu _r$. The envelope of the following nine combinations is considered: (ISR $\mu _r/p_{{\mathrm{T}}}$ , FSR $\mu _r/p_{{\mathrm{T}}}$) $= (0.5,0.5)$, (0.5, 1), (0.5, 2), (1, 0.5), (1, 1), (1, 2), (2, 0.5), (2, 1), and (2, 2).

The nonperturbative contributions (MPI and hadronization) are included in the calculations above. The uncertainty from these contributions are estimated from the different choices of the UE tune and found to be negligible.

The uncertainty from PS dominates for the normalized inclusive 2-jet distributions. It is one order of magnitude larger than the rest of the sources near $\varDelta \phi _{12} =180^{\circ }$. On the other hand, for the normalized inclusive 3-jet distributions, the main contributions come from PS and PDF uncertainties. The predictions from ph-2j + pythia 8 and ph-2j + herwig++ (Fig. 3) show the differences from using different PS models together with different matching procedures.

Figure 7 (8) show the ratios of the ph-2j predictions to data for the normalized inclusive 2(3)-jet distributions for the different $p_{{\mathrm{T}}} ^{\mathrm{max}}$ regions. The solid beige band indicates the total experimental uncertainty, and the hatched band represents the total theoretical uncertainty.

For the inclusive 2-jet distributions, the theoretical uncertainty is larger than the experimental one in the region close to $\varDelta \phi _{12} =180^{\circ }$ (Fig. 7). This is because the contribution from PS dominates in this region, and its uncertainty is large. For the inclusive 3-jet distributions (Fig. 8), the theoretical uncertainty is smaller in the region close to $180^\circ$. In this case, the region close to $180^\circ$ is not filled by the partons from the PS, but by the third parton from ph-2j, leading to a smaller PS uncertainty.

## Summary

Measurements of the normalized inclusive 2- and 3-jet distributions as a function of the azimuthal separation $\varDelta \phi _{12}$ between the two jets with the highest transverse momentum $p_{{\mathrm{T}}}$, in the collinear back-to-back region, are presented for several $p_{{\mathrm{T}}} ^{\mathrm{max}}$ ranges of the leading jet. The measurements are performed using data collected with the CMS experiment at the LHC, corresponding to an integrated luminosity of 35.9$\,\text {fb}^{-1}$ of p p collisions at a center-of-mass energy of $13\,\text {Te}\text {V}$.

The measured $\varDelta \phi _{12}$ distributions generally agree with predictions from pythia 8, herwig++, MadGraph + pythia 8, ph-2j + herwig++, and powheg (ph-2j and ph-3j) matched to pythia 8. Discrepancies between the measurement and theoretical predictions are as large as 15%, mainly in the region $177^\circ< \varDelta \phi _{12} < 180^\circ$. The predictions agree better with the measurements for larger $p_{{\mathrm{T}}} ^{\mathrm{max}}$ and smaller $\varDelta \phi _{12}$, where the contribution of resummation effects becomes smaller. The 2- and 3-jet measurements are not simultaneously described by any of models.

The tree-level multijet event generator MadGraph in combination with pythia 8 for showering, hadronization, and multiparton interactions, shows deviations from the measured $\varDelta \phi _{12}$ for the inclusive 2-jet case, and even larger deviations for the 3-jet case. The pythia 8 and herwig++ predictions show deviations (up to 10%) for the 2-jet inclusive distributions, whereas their predictions are in reasonable agreement with the inclusive 3-jet distributions.

The next-to-leading-order ph-2j + pythia 8 prediction does not describe the data and a different trend compared to pythia 8 and herwig++ towards $\varDelta \phi _{12} =180^{\circ }$ is observed. The ph-3j + pythia 8 predictions agree with the measurements except for the last bin in the low $p_{{\mathrm{T}}} ^{\mathrm{max}}$ intervals. The ph-2j + herwig++ prediction agrees well with the measurement in the highest $p_{{\mathrm{T}}} ^{\mathrm{max}}$ ranges. For the inclusive 3-jet case, ph-2j + pythia 8 performs similarly to pythia 8 and herwig++ in the whole $\varDelta \phi _{12}$ range for high $p_{{\mathrm{T}}} ^{\mathrm{max}}$ intervals. MadGraph + pythia 8, ph-3j + pythia 8, and ph-2j + herwig++ show deviations from the measurements of up to 15%.

The measurement of correlations for collinear back–to–back dijet configurations probes the multiple scales involved in the event and, therefore, the differences observed between predictions and the measurements illustrate the importance of improving the models of soft parton radiation accompanying the hard process.

## Data Availability

This manuscript has no associated data or the data will not be deposited. [Authors’ comment: Release and preservation of data used by the CMS Collaboration as the basis for publications is guided by the CMS policy as written in its document “CMS data preservation, re-use and open access policy” (https://cms-docdb.cern.ch/cgi-bin/PublicDocDB/RetrieveFile?docid=6032&filename=CMSDataPolicyV1.2.pdf&version=2).]
